# The weaponization of artificial intelligence: What the public needs to be aware of

**DOI:** 10.3389/frai.2023.1154184

**Published:** 2023-03-08

**Authors:** Birgitta Dresp-Langley

**Affiliations:** UMR 7357 CNRS, Université de Strasbourg, Strasbourg, France

**Keywords:** artificial intelligence, weaponization, autonomous weapon systems, the Geneva protocol, the discrimination principle, Just War Theories, *adversarial hacking*, European AI Act

## Abstract

Technological progress has brought about the emergence of machines that have the capacity to take human lives without human control. These represent an unprecedented threat to humankind. This paper starts from the example of chemical weapons, now banned worldwide by the Geneva protocol, to illustrate how technological development initially aimed at the benefit of humankind has, ultimately, produced what is now called the “Weaponization of Artificial Intelligence (AI)”. Autonomous Weapon Systems (AWS) fail the so-called discrimination principle, yet, the wider public is largely unaware of this problem. Given that ongoing scientific research on AWS, performed in the military sector, is generally not made available to the public domain, many of the viewpoints on this subject, expressed across different media, invoke common sense rather than scientific evidence. Yet, the implications of a potential weaponization of our work as scientists, especially in the field of AI, are reaching further than some may think. The potential consequences of a deployment of AWS for citizen stakeholders are incommensurable, and it is time to raise awareness in the public domain of the kind of potential threats identified, and to encourage legal policies ensuring that these threats will not materialize.

On the first of May in 1915, Clara Haber (Friedrich and Hoffmann, 2016), née Immerwahr, committed suicide. A week before her death, her husband, the German scientist Fritz Haber (Witschi, 2000), had organized the first chlorine-gas attack at Ypres in Belgium, which was aimed at breaking the military stalemate in Germany's favor. Ten years before, in 1905, Haber had achieved what other peers before him had attempted in vain. Using high pressure and a catalyst, Haber was able to trigger a direct reaction between nitrogen gas and hydrogen gas to create ammonia. The process is considered as one of the most important technological breakthroughs of the 20th century as it enabled the mass production of agricultural fertilizers supporting half of the world's food base and leading to a massive increase in the growth of crops for human consumption. During the First World War, Haber developed a new weapon, poison gas (the first of which was chlorine gas) and supervised its initial deployment on the Western Front at Ypres in Belgium and thereby became “the father of chemical warfare” (Fitzgerald, 2008), which is believed to have prompted in 1915 the suicide of his wife, herself a chemist. In 1918, Haber was awarded the Nobel Prize in Chemistry for the synthesis of ammonia from its elements. In the world of today, chemical weapons are considered weapons of mass destruction, and their use in armed conflict is a violation of international law. The Chemical Weapons Convention (2021), ratified by 145 nations and in effect since 1997, strictly prohibits the production, storage, or use of toxic chemicals as weapons of war. Chemical weapons are weapons of mass destruction, and a zero tolerance policy for these at the international level is stipulated in the Geneva Protocol of the United Nations (The United Nations Office for Disarmament Affairs, 1925). Recent scientific developments in the fields of organic synthesis and chemical design (Lei et al., 2019; Deng et al., 2020), however, now pose challenges to these conventions and may compromise their continued implementation in the future. In addition, technological progress in the field of Artificial Intelligence (AI) has brought about the emergence of machines that have the capacity to take human lives without human control (Burton and Soare, 2019), with the possibility of combining innovation in chemical design with robots controlled by AI. This has potentiated the manufacturing and deployment of an entirely new breed of autonomous weaponry, representing unprecedented threats to humanity, for which there is currently no legal framework (Armitage, 2019). The risk of an emergence of novel forms of weapons of mass destruction under a radically different, more sophisticated and pernicious, form has now become real. New maximum-risk weapons of mass destruction could be drone swarms and autonomous CBRN (Chemical, Biological, Radiological, Nuclear) weapons, which include miniature insect drones reduced to undetectable devices capable of administering lethal biochemical substances through their stings. Science and society are challenged by such unprecedented technological development as it brings about changes where clear lines dividing fundamental science from application, benefits from risks, and responsible deployment from abuse can no longer be drawn. For science and research in AI, the core problem here is that of responsible technological development. Autonomous Weapon Systems (AWS) have been announced to the public as the third revolution in warfare (Reports From the American Association for the Advancement of Science Meeting in Washington DC, 2019). The implications of this revolution for humanity, or the fundamental questions it raises with regard to responsible technological development in the fields of AI, robotics, drones, or autonomous vehicles and platoons are, however, not so widely discussed in the public domain. Although not systematically extended to the deployment of AWS, the design principles, algorithms, and technology produced in any of these fields in science can be directly translated into a novel solution for further development of AWS. This has brought about one the most pressing current problems in science and technology. Ethical insights and debate are useful and necessary, but unlikely to bring about the urgently required solutions for minimizing the associated risks. Various kinds of autonomous weapon systems (AWS) are already out there, i.e., currently being developed and/or already employed. Their different levels of autonomy will be briefly summarized here. This will be followed by a brief explanation of the “just” vs. “unjust wars” dilemma in ethics (Walzer, 1977; McMahan, 2007) and, finally, a summary of the risks of autonomous weaponry for humankind and the ensuing need for policy making at the international level.

The earliest example of an autonomous vehicle was The American Wonder developed in 1925 (Kröger, 2016), which cruised the streets of New York City remotely controlled by another vehicle following behind, an early demonstration of platooning (Maiti et al., 2017), i.e., the coordinated formation of vehicles navigating in a fleet under shared automatic control. Since then, advancements in technology enabled functionalities like adaptive and predictive cruise control combined with RADAR, LIDAR, high-resolution 360-degree cameras and, ultimately, AI (Maiti et al., 2017). Scientists and experts have begun to raise their voices against the dangers associated with these technological developments and their weaponization for humankind (Carriço, 2018; Khakurel et al., 2018; Di and Shi, 2021). Lethal autonomous weapons and AWS currently exploiting AI, under development and/or already employed, include autonomous stationary sentry guns and remote weapon stations programmed to fire at humans and vehicles, killer robots (also called “slaughter bots”), and drones and drone swarms with autonomous targeting capabilities.

## Autonomous stationary sentry guns

A sentry gun is a remote weapon that is automatically aimed and fired at targets that are detected by sensors. The earliest functioning military sentry guns were close-in point-defense weapons used for detecting and destroying short range incoming missiles and enemy aircraft. Such were first used exclusively on ships, but are now also land-based defenses. The first of its kind to have an integrated system that includes surveillance, tracking, firing, and voice recognition would be the SGR-A1, jointly developed by Hanwha Aerospace and Korea University to assist South Korean troops in the Korean Demilitarized Zone in a highly classified project.

## Autonomous killer robots

Killer robots or “slaughter bots”, are autonomous robotic systems able to select and attack targets without intervention by a human operator (Righetti et al., 2014). While in some of these systems, the initial command to attack would be given by a human and the robot then has a degree of autonomous “choice” for action, other systems without any human in the loop are currently tested in several countries. Therein, the decision to deploy lethal force is delegated to a machine. Such far-reaching development would fundamentally change warfare of the future. The function of autonomously selecting and attacking targets could be applied to various platforms such as battle tanks, fighter jets, or ships. Another term used to describe these weapons is lethal autonomous weapon systems (LAWS). When equipped with advanced sensors and AI, moreover, autonomous weapons could be trained to operate in coordinated platoons to overwhelm enemy defenders, in distributed surface-warfare action groups or electronic warfare vessels, all unmanned and operating autonomously.

## Autonomous drones and swarms

In October 2013, the United States Strategic Capabilities Office launched 103 Perdix drones, which communicated using a “distributed brain” to assemble into a complex formation, travel across a battlefield, or regroup into a new formation. The swarm was created by MIT engineering students using commercially available components and design. In theory, drone swarms could be scaled to tens of thousands of drones to create an autonomous weapon akin to a low-scale nuclear device (Müller, 2016). Armed, fully-autonomous drone swarms are deemed to become future weapons of mass destruction because they combine two properties unique to traditional weapons of mass destruction: mass harm and lack of human control to ensure the weapons do not harm civilians. Experts doubt that any single autonomous weapon could ever be capable of adequately discriminating between civilian and military targets, and with thousands or tens of thousands of drones is a swarm, this risk becomes incommensurable (Kallenborn, 2021).

In summary, AWS are lethal devices that identify potential enemy targets and independently choose to attack those targets on the basis of algorithms and AI. AWS other than stationary sentry guns require the integration of several core elements: a mobile combat platform, sensors of various types to scrutinize the platform's surroundings, a processing systems to classify objects discovered by the sensors, and algorithms that prompt the system to initiate attack when an allowable target is detected. The U.S. Department of Defense described an autonomous weapons system as a “weapons system that, once activated, can select and engage targets without further intervention by a human operator” (Scharre, 2016). While there is currently no international consensus on a definition of AWS, they have been rated according to the level of their autonomy from human control. The concept of autonomy in the context of AWS may be defined as the ability of the system to execute a task or set of operations without human input through action upon or interaction with its environment that are determined and controlled by algorithms. What matters critically to the definition of an AWS appears to be the type of decision or function that is rendered autonomous by no longer being under the control of a human operator. Under this premise, three levels of increasing autonomy may be proposed for AWS (Kallenborn, 2021):

- Supervised autonomous weapon or “human on-the-loop” system, is autonomous weapon system that is designed to provide human operators with the ability to intervene and terminate engagements before unacceptable levels of damage occur. Examples would include defensive weapon systems used to attack, which would independently select and attack targets according to their program while a human retains the full supervision of all operations and can override the system, if necessary, within a limited time-period.
- Semi-autonomous weapon or “human-in-the-loop” system, once activated, is intended to only engage individual targets or specific target groups that have been selected by a human operator. Examples would include homing munitions that, once launched to a particular target location, search for and attack preprogrammed categories of targets within the area.
- Fully autonomous weapon or “human out-of-the-the-loop” system, once activated, can select and engage targets without further intervention by a human operator. Examples would include “loitering” weapons that, once launched, search for and attack their intended targets over a specified area without any further human intervention, or weapon systems that autonomously use electronic “jamming” to disrupt communications.

Some of the critical functions of such weapon systems have been automated for many years. A weapon system does not necessarily need to be highly complex to be autonomous, which is illustrated by existing anti-personnel weapon systems that have autonomous modes such as the so called sentry guns (cf. see here above). Autonomous weapon systems in use today, autonomous, semi-autonomous, or supervised according to the definitions provided here above, are claimed to be constrained in several respects (Righetti et al., 2014; Scharre, 2016; Kallenborn, 2021). First, they are claimed to be limited in the tasks they are employed for, with defensive action against rocket attacks, or offensive action against specific military installations such as radar, for example. Second, they are claimed to be limited in the types of targets they attack, which are reduced to vehicles or objects rather than civilians. Third, they are claimed to be used in relatively simple and predictable environments such as at high sea, or on land areas that are remote from populated zones. However, the potential of AWS to become weapons of mass destruction is real, and scientists, experts, and journalists worldwide are expressing concern about the fundamentally unethical nature of the development and/or deployment of AWS (U.S. Department of Defense, 2021). From an ethical standpoint, AWS are not eligible whatever their level of autonomy, as they all fail in satisfying the *principle of discrimination* stipulated in the framework of contemporary military ethics under the premise of Just War Theories (Walzer, 1977; McMahan, 2007).

Autonomous Weapon Systems raise many questions and concerns. Addressing them requires a multidisciplinary research effort on the one hand, and public discussions on ethical and moral responsibility on the other. While ethical standards for decision-making are to some extent studied in relationship with the research and development of autonomous vehicles and human operated drones, such have not yet widely been extended to AWS (Brough et al., 2007; Müller, 2016; Scharre, 2016; Kallenborn, 2021; U.S. Department of Defense, 2021). In fact, proposing ethical standards for moral judgment or decision making on the development and/or deployment of AWS requires taking into account ethical rules of warfare as such. In the history of cultures and society, Saint Augustine was the first individual in Christianity to have proposed a theory on war and justice, the so-called Just-War Theory. He referred to the Bible and claimed that some wars are necessary to fight evil. Saint Thomas Aquinas revised Augustine's theory and proposed several criteria for a just war: it needs to be waged by a legitimate authority, have a just cause, follow the right intentions, have a reasonable probability of success, the nations involved in the war must avoid disproportionate military action, only use the amount of force absolutely necessary, and the use of force must distinguish between the militia and civilians. This last principle is called the *principle of discrimination* in contemporary military ethics (Guersenzvaig, 2018). It is to ensure that innocent citizens do not become the target of war, and that the killing of civilians is avoided at all cost. Just-War Theory in contemporary ethics builds on these principles as a set of rules for military combat where conventions are meant to serve as guides to human action. While true blue pacifists reject war in any form as immoral, and thereby imply that all acts within war are immoral and inexcusable and true bleu militarists believe that in war “all is fair”, just war theorists take the pragmatic stance that, should war break out for one reason or another, considerations relative to its justification are necessary, and rules and procedures need to be followed to ensure that specific sanctuaries from war's dreadful consequences are upheld and protected. Contemporary just war theory (Brough et al., 2007) concludes that the use of autonomous technologies is neither completely morally acceptable, nor is it morally unacceptable (Guersenzvaig, 2018; Armitage, 2019; Reports From the American Association for the Advancement of Science Meeting in Washington DC, 2019). Any technology of warfare could be just or unjust depending on the situation because what is and is not acceptable in war is ultimately a *convention*. However, while such theories extrapolate from the conventions proposed by Saint Augustine and Saint Thomas Aquinas in an attempt to deal with new technologies like AWS, they remain mere speculation. Also, the principles of ethical warfare in Just War Theory “non-negotiable”, i.e., when one of these principles is violated by a procedure of a type of weapon, the ethical debate regarding the latter is, in principle, settled. The major ethical objection against AWS is the fact that, whatever their level of autonomy, they all fail the *principle of discrimination* in the sense that one cannot ensure that they will not harm civilians (Guersenzvaig, 2018). Therefore, the case of AWS belongs into the realm of international law and policy making, and it is up to the international community to establish a new set of conventions to regulate their use through international legislation and treaties. Such a process can be informed by ethics theory to clarify the moral foundations for AWS control under the light of individual rights or other solid moral grounds. However, while ethical theory might positively influence the practical control of this technology through international law, an ethical debate *per se* cannot solve the problem of the many threats AWS represent for humankind. In addition to these threats, our planet is running out of resources. Wars (whatever form they may take) are expensive. Governments urgently need to focus on technological development for sustainability instead of wasting precious resources on new types of weaponry that, beyond failing the principle of discrimination, are unsafe in other aspects. There is no such thing as an autonomous system that cannot be hacked, and the risk that non-state actors take control of AWS through adversarial hacking is real. In areas from robotics and AI to the material and life sciences, the coming decades could bring about innovation and scientific progress that should help us promote peace, protect our planet, and resolve the root causes of poverty and suffering worldwide. With the ability to interact through cyberspace to spread and exchange information, and to reinforce technological development for peace and sustainability in an increasingly networked world, this goal is severely jeopardized by adversity from various sources. Should war break out, failure of AWS, whatever their level of autonomy, to satisfy the *principle of discrimination* as a major threat is compounded by other risks that lead to argue for banning the development and/or the deployment of AWS by law (Boulanin and Verbruggen, 2017; Russell et al., 2021). The deployment of AWS can pose difficulties for the attribution of hostile acts and lead to unintentional escalation of conflicts. Moreover, non-state actors such as terrorist groups and international criminal networks could harness or sabotage the technology in service of their own agendas through what is called *adversarial hacking*. This risk is real (Edgar and Manz, 2017) and concerns AWS with any level of autonomy (cf. chapter 3 here above), including “human-on-the-loop” or supervised autonomous systems, that can operate independently but are under the oversight of a human who is supposed to intervene if “something goes wrong”.

In its simplest definition, *adversarial hacking* is an action with malicious intent performed by someone or a group to compromise a system or the cyber resources used by that system. The US Defense Science Board Task Force Report on Resilient Military Systems and Advanced Cyber Threat (U.S. Department of Defense, 2012) divides potential sources of adversarial attacks (*adversaries*) into three major categories:

1. Adversaries using off-the-shelf tools that exploit system vulnerabilities.
2. Adversaries with resources and capabilities to discover new, unsuspected vulnerabilities.
3. Adversaries that can invest billions of dollars and unlimited time for the development of new tools to create new vulnerabilities.

One may not be able to imagine the amount of resources that category three adversaries could deploy for attacks that impact the cyber capabilities of any AWS. Attacks by *adversarial hacking* can target any level of such systems, from the infrastructures that records/measures state information, to the algorithms and processes that govern the automatic control systems, whether supervised by human operators or not. Sentient adversaries to the system may act to corrupt state information, interrupt communications, or to modify the automatic control systems of AWS. This could modify the dynamics and/or the structure of their entire physical network. The adversaries may be then able to access and corrupt both local and network-wide state information, and to cause local or network-wide perturbations to the physical network. The results of *adversarial hacking* could generate an unknown variety of different types of attack on an AWS system causing, in the best case scenario system failure, or producing scenarios where the system is corrupted to do what it is not supposed to (i.e., kill civilians, for example).

History has many examples where scientific progress and innovation initially aimed at humankind's benefit was then applied for warfare. Scientific insights into biological modification and synthesis designed to help scientists better understand disease could be misused to increase the potency of infectious agents deployed by AWS. Furthermore, such weapons raise serious concerns about their potential misuse by non-state actors. The cyberspace delivers the critical infrastructure for AWS, yet, it is not a safe place. In his 2017 book, Tegmark (2017) discussed the implications of warfare by AI, and how science can ensure that we keep AI beneficial to humankind. In the meantime, however, the weaponization of AI has become real and has produced AWS, some of which function without any human in the loop, which could have unintended, unforeseen, and unprecedentedly devastating consequences for humankind. A first step toward limiting the weaponization of AI by development or deployment of lethal autonomous weapons is to increase awareness in the public domain. The scientific community will have to assume responsibility in this process. Novel forms of warfare by AI-controlled weaponry with a potential for mass destruction threaten the continued effectiveness of existing conventions such as the Geneva Protocol. This has produced a new dilemma that cannot be resolved by ethical debate, only by international law and policy making. A European AI strategy has been carved out in a white paper by The European Commission (2020). The strategy aims at ensuring that AI is human-centric and trustworthy. Such an objective translates into the European approach to excellence and trust through concrete rules and actions as stipulated in the AI Act where the Commission and EU Member States present its AI package (with EU Member States) with a proposal for a regulatory and harmonized rules on AI with relevant impact assessment. The Artificial Intelligence Regulation Act of the European Commission (European AI Act) is the first to propose an attempt toward a transnational legal framework on AI. It assigns applications of AI arbitrarily to three categories of a risk pyramid, with many loopholes and exceptions. Applications not explicitly listed under “high-risk” are largely left unregulated, including autonomous weapons systems. Such shortcomings limit the Act's ability to ensure AI as a force for good in citizens lives. In addition, the law is in many ways inflexible. If, for example, in a few years' time a non-listed but dangerous AI application is used in an unforeseen sector, such as the military, the law provides no mechanism to label it as “high-risk”. Following multiple amendments and discussions, the EU Member States and the Council of the EU approved a compromised version of the initially proposed Artificial Intelligence Regulation (AI Act) on December 6, 2022. This is only as small first step in what promises to be a long process of recognition for international policies beyond Europe. The Geneva Convention stems from the need for promoting regional and international peace and security, and to free the world from the scourge and burden of weapons of mass destruction. While it recognizes the need for a comprehensive approach toward weapons in a balanced and non-discriminatory manner as a contribution to international peace and security, there are currently no instruments of ratification concerning artificial intelligence for the emerging breed of autonomous weapons and their deployment to the service of war.

## Data availability statement

The original contributions presented in the study are included in the article/supplementary material, further inquiries can be directed to the corresponding author.

## Author contributions

The author confirms being the sole contributor of this work and has approved it for publication.
